# Case report: Regression of in-transit metastases of cutaneous squamous cell carcinoma with combination pembrolizumab and topical diphencyprone

**DOI:** 10.3389/fonc.2024.1294331

**Published:** 2024-05-10

**Authors:** Dina Poplausky, Jade N. Young, Brandon R. Block, Yeriel Estrada, Giselle K. Singer, Vicky Wong, Patricia Cabral, Yamato Suemitsu, Randie H. Kim, Philip Friedlander, Nicholas Gulati

**Affiliations:** ^1^ Department of Dermatology, Icahn School of Medicine at Mount Sinai, New York, NY, United States; ^2^ Department of Pathology, Icahn School of Medicine at Mount Sinai, New York, NY, United States; ^3^ Division of Hematology and Medical Oncology, The Tisch Cancer Institute, Icahn School of Medicine at Mount Sinai, New York, NY, United States

**Keywords:** cutaneous metastases, metastatic squamous cell carcinoma, diphencyprone (DCP, DPCP), immune check inhibitor (ICI), open-label clinical study, phase 1 cancer trials

## Abstract

While typically low-risk, cutaneous squamous cell carcinoma (cSCC) can infrequently progress to metastatic disease with in-transit lesions, localized to the dermis or subcutaneous tissue between the primary tumor and draining regional lymph nodes. These lesions are associated with poor prognostic values, including decreased survival rates and increased risk of recurrence. We present the case of a 75-year-old male with cSCC and in-transit metastases on his scalp treated with the immune checkpoint inhibitor (ICI) pembrolizumab in conjunction with diphencyprone (DPCP), a topical hapten that induces a delayed-type hypersensitivity reaction in the skin. The patient was enrolled in a clinical trial (NCT05481658) that involved the twice-weekly application of DPCP 0.04% ointment to four of the in-transit metastases on his frontal scalp, concurrent with pembrolizumab 300 mg administered every three weeks. Following effective sensitization and a twelve-week treatment course, complete clearance of all lesions, DPCP-treated and non-DPCP treated, was achieved, with no adverse events. The immunologic profiles of the post-treatment biopsies were analyzed by TaqMan Low Density Array quantitative real-time polymerase chain reaction to measure immune marker gene expression. Relative to the non-DPCP-treated lesion, the DPCP-treated lesion demonstrated increased pro-inflammatory genetic markers and T-cell activation. This case represents the first reported instance of in-transit metastases of cSCC successfully treated with DPCP and an ICI. It highlights the potential safety and efficacy of DPCP with systemic immunotherapy for the management of in-transit metastases of cSCC in patients for whom surgery and radiation may be contraindicated.

## Introduction

Cutaneous squamous cell carcinoma (cSCC) accounts for about 20% of all skin cancers and has a rising incidence in the United States (1). Although typically low-risk, 1.2-5% of cSCCs metastasize (2). In-transit metastases are defined as distinct lesions originating in the dermis or subcutaneous tissues occurring before the first station of regional lymph nodes. Prior research on cutaneous metastasis is limited; however, they typically confer poor prognosis and are a manifestation of end-stage disease (3). A multicentric cohort study in March 2023 found that the size and number of in-transit metastases are associated with an increased risk of relapse, and the number of in-transit metastases is associated with decreased survival (4).

Treatment often includes a combination of surgery, radiation, and chemotherapy or immunotherapy. However, despite aggressive treatment options available, one-year survival is 45-69% (3). Immune checkpoint inhibitors, including programmed death 1 (PD-1) and programmed death ligand 1 (PD-L1) inhibitors, are increasingly used in various cancers. The Food and Drug Administration (FDA) approved the PD-1 inhibitors pembrolizumab and cemiplimab for the treatment of cSCCs not curable by surgery or radiation therapy. Pembrolizumab demonstrated a complete response (CR) rate of 10.5% and a partial response (PR) rate of 24.8% for patients with recurrent or metastatic cSCC. Cemiplimab demonstrated a CR rate of 6.8% and a PR rate of 40.7% in patients with metastatic cSCC (5).

Here we report the case of a 75-year-old male with in-transit metastases of cSCC treated with diphencyprone (DPCP) ointment and pembrolizumab.

## Case Description

**Figure 1:**
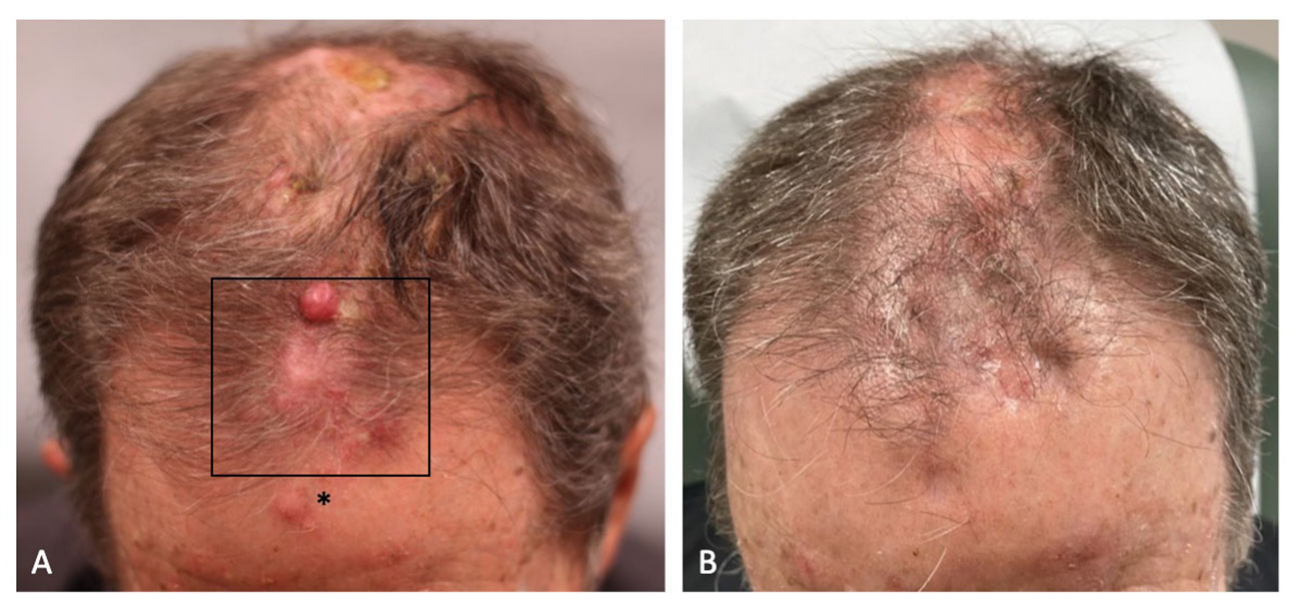
**(A)** The patient’s frontal scalp demonstrated erythematous nodules, consistent with in-transit metastases of cutaneous squamous cell carcinoma, before combination treatment with pembrolizumab and diphencyprone (DPCP). DPCP was applied to the boxed area. The asterisk denotes the area of sensitization. **(B)** Regression of the in-transit metastases after a 12-week course of combination treatment with pembrolizumab and DPCP.

A 75-year-old male with an extensive history of keratinocyte carcinomas presented to an outside dermatologist with a new scalp lesion. The clinical examination was notable for an ulcerated, erythematous indurated plaque on the vertex scalp. A biopsy was performed which revealed invasive cSCC, poorly differentiated with positive deep and peripheral margins, and a thickness of 5.5 mm. The patient then underwent Mohs micrographic surgery, and the tumor was removed in two stages. The frozen section pathology demonstrated aggregates of atypical keratinocytes in the dermis and subcutaneous tissue. The patient opted to heal by secondary intention. Two months after the primary tumor resection, the patient presented with multiple, firm, erythematous nodules on his scalp (**Figure 1A**). Biopsies of the right and left crown of the scalp revealed poorly differentiated cSCC representing in-transit metastases.

Given the extent of his multifocal disease, he was not a candidate for surgical resection or radiotherapy and was therefore referred to medical oncology for systemic treatment. The workup included a positron emission tomography (PET) scan, which demonstrated multiple hypermetabolic soft tissue nodules throughout the scalp, compatible with biopsy-proven cSCC. There was no evidence of internal metastases. The patient began pembrolizumab 300 mg every three weeks as part of standard-of-care treatment. He was simultaneously enrolled in a clinical trial using DPCP ointment in conjunction with PD-1 or PD-L1 immune checkpoint inhibition for the treatment of cancer patients with cutaneous metastases (ClinicalTrials.gov identifier NCT05481658).

**Figure 2:**
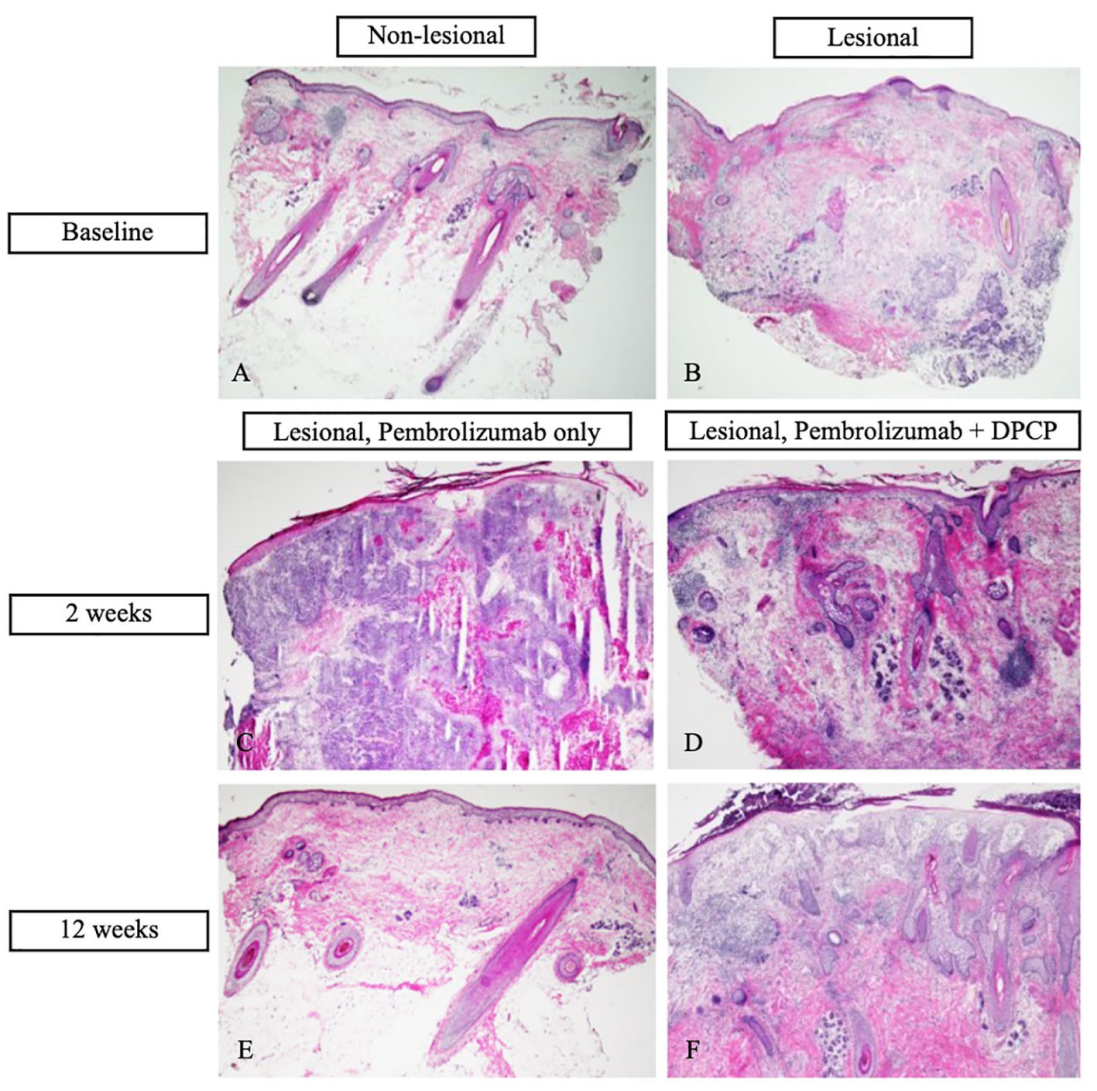
Hematoxylin and eosin (H&E) stains of cutaneous biopsies. **(A)** Baseline non-lesional skin demonstrates benign skin tissue, negative for tumor (20X magnification). **(B)** Baseline in-transit metastasis demonstrates moderately to poorly differentiated infiltrative cutaneous squamous cell carcinoma (cSCC) with focal keratinization completely within the dermis. There is mild inflammatory infiltrate associated with tumor proliferation (20X). **(C)** In-transit metastasis two weeks post-treatment with pembrolizumab only (diphencyprone (DPCP) not applied) demonstrates diffuse dermal infiltration of moderately to poorly differentiated cSCC with focal keratinization (20X). **(D)** In-transit metastasis two weeks post-treatment with DPCP and pembrolizumab demonstrates lichenoid dermatitis with diffuse dermal inflammatory and fibrotic changes. No cSCC is identified (40X). **(E)** In-transit metastasis after 12 weeks of treatment with pembrolizumab only demonstrates benign skin tissue with minimal changes including mild dermal fibrosis and perivascular inflammatory infiltrate (20X). **(F)** In-transit metastasis after 12 weeks of treatment with DPCP and pembrolizumab demonstrates spongiosis, superficial dermal edema, patchy inflammatory infiltrate, and dermal fibrosis. No cSCC is identified (40X).

Cutaneous punch biopsies of lesional and non-lesional tissue were collected at baseline before systemic and topical oncologic therapy for hematoxylin and eosin (H&E) staining (**Figures 2A, B**) and were analyzed by TaqMan Low Density Array quantitative real-time polymerase chain reaction (TLDA qRT-PCR) to measure immune marker gene expression. The patient was sensitized to 0.4% DPCP on one of his in-transit metastases and his right upper arm, as well as to 0.04% DPCP on his left lower arm. Effective sensitization was confirmed two weeks later by notable erythema and scaling. The patient was then treated with DPCP 0.04% ointment twice weekly for 12 weeks, applied to four in-transit metastases on the frontal scalp. This topical treatment was started concurrently with pembrolizumab. Five in-transit metastases were not treated with DPCP.

After two weeks of dual treatment with pembrolizumab and DPCP, regression was observed for the DPCP-treated metastases only. Given the positive clinical response, additional cutaneous biopsies were performed of a DPCP-treated metastasis and a non-DPCP-treated metastasis (**Figures 2C, D**). At the end of the 12-week treatment period, the patient had resolution of all metastases, including the DPCP-treated and non-DPCP-treated lesions (**Figure 1B**). At this time, follow-up biopsies of a prior DPCP-treated metastasis and a non-DPCP-treated metastasis were taken (**Figures 2E, F**). The patient has continued pembrolizumab per the treating oncologist. At the time of publication, the patient is on month 18 of pembrolizumab treatment. A repeat PET scan demonstrated a reduction in the metabolic activity of the previously noted soft tissue lesions in the scalp, suggestive of a favorable tumor response. The patient was monitored for adverse events at each twice-weekly visit. The side effects of DPCP included expected erythema, skin irritation, and mild pruritus, all of which were tolerable to the patient and managed with conservative treatment. There were no DPCP drug interruptions due to toxicities. At the patient’s most recent follow-up four months after his last DPCP treatment application, the patient remains clinically clear of cutaneous metastases.

The immunologic profiles of the post-treatment biopsies were characterized by TLDA qRT-PCR. Relative to the non-DPCP-treated lesion, the DPCP-treated lesion demonstrated upregulation of *IL-2*, *ICOS*, and *PD-1* by 4.96-fold, 4.31-fold, and 10.70-fold, respectively. Moreover, *CXCL1* and *IL-1B* gene expression were upregulated by 4.13-fold and 3.03-fold, respectively.

## Discussion

This is a case of a patient who achieved resolution of in-transit metastases of cSCC with DPCP and an ICI. To date, there is no reported use of topical treatments concurrent with systemic therapy for in-transit metastases of cSCC. Given that survival rates for end-stage cSCC are low, new treatment paradigms must be explored.

DPCP is a topical hapten that induces a delayed-type hypersensitivity reaction in the skin. It has historically been employed for the treatment of warts (6), but has more recently shown efficacy in the treatment of cutaneous metastases of melanoma (7). Of note, DPCP has also been used successfully in conjunction with an ICI for cutaneous metastases of melanoma (8).

DPCP has been shown to increase both *PD-1* gene expression in tissue and PD-1 protein levels in the serum (9). By increasing the PD-1 targets, DPCP may amplify the inhibition induced by pembrolizumab (9), thus allowing for synergistic anti-tumor immune activation. Clinically and histopathologically, the patient’s DPCP-treated metastases demonstrated earlier regression in comparison to those treated with ICI alone. Therefore, the addition of a topical immunotherapy agent to systemic immunotherapy may be more effective than systemic monotherapy. While all lesions, both DPCP-treated and non-DPCP-treated, ultimately resolved in the setting of pembrolizumab, the earlier resolution of lesions with DPCP demonstrates the synergy with pembrolizumab and may have positive clinical implications for patients. Prior research suggests that DPCP is not systemically absorbed (10); however, the immune response to DPCP may affect areas not topically treated with DPCP (8, 11).

The comparison of non-DPCP-treated lesion and the DPCP-treated lesion isolates and quantifies DPCP synergism with a systemic ICI. *IL-2* and *ICOS* are markers for T-cell activation (12, 13), which may contribute to its anti-tumor effect (14). In line with the current literature, *PD-1* was upregulated in the DPCP-treated lesion, thereby augmenting the substrate available for pembrolizumab. Moreover, the upregulation of *CXCL1* and *IL-1B* demonstrates increased activation of pro-inflammatory genetic markers (15, 16).

The patient tolerated the combination therapy well with minimal, expected skin inflammation. This topical modality may be particularly useful in patients for whom surgery or radiation may be contraindicated. Given that in-transit metastases are often a sign of terminal disease, patients may not be able to tolerate more aggressive treatments. Additionally, DPCP is an inexpensive therapy (17), having positive economic impacts on both patients and the healthcare system.

## Conclusion

We describe, to our knowledge, the first case of in-transit metastases of cSCC treated successfully with DPCP and a PD-1 inhibitor, potentially due to synergistic immune activation. Our results indicate that, in this patient, DPCP was an effective and safe treatment in conjunction with systemic immunotherapy. Further exploration with long-term data and a larger patient population is needed to evaluate the safety, efficacy, and clinical utility of DPCP in conjunction with an ICI for in-transit metastases of cSCC.

## Data availability statement

The raw data supporting the conclusions of this article will be made available by the authors, without undue reservation.

## Ethics statement

The studies involving humans were approved by Human Research Protection Program at the Icahn School of Medicine at Mount Sinai (ISMMS). The studies were conducted in accordance with the local legislation and institutional requirements. The participants provided their written informed consent to participate in this study. Written informed consent was obtained from the individual(s) for the publication of any potentially identifiable images or data included in this article.

## Author contributions

DP: Conceptualization, Data curation, Formal analysis, Investigation, Methodology, Project administration, Resources, Supervision, Validation, Writing – original draft, Writing – review & editing. JY: Conceptualization, Data curation, Formal Analysis, Investigation, Methodology, Project administration, Resources, Supervision, Validation, Writing – original draft, Writing – review & editing. BB: Investigation, Project administration, Writing – original draft, Writing – review & editing, Data curation, Formal Analysis. YE: Data curation, Formal analysis, Investigation, Resources, Writing – review & editing. GS: Resources, Supervision, Writing – review & editing. VW: Resources, Supervision, Writing – review & editing. PC: Project administration, Supervision, Writing – review & editing. YS: Formal analysis, Resources, Writing – review & editing. RK: Formal analysis, Resources, Writing – review & editing. PF: Resources, Supervision, Writing – review & editing. NG: Conceptualization, Data curation, Formal analysis, Funding acquisition, Investigation, Methodology, Project administration, Resources, Supervision, Validation, Writing – review & editing.
